# Paradox and context shift

**DOI:** 10.1007/s11098-022-01841-2

**Published:** 2022-07-05

**Authors:** Poppy Mankowitz

**Affiliations:** University of Bristol, Bristol, United Kingdom

**Keywords:** Liar paradox, Contextualism, Context shift, Natural language

## Abstract

The Liar sentence *L*, which reads ‘*L* is not true’, can be used to produce an apparently valid argument proving that *L* is not true and that *L* is true. There has been increasing recognition of the appeal of contextualist solutions to the Liar paradox. Contextualist accounts hold that some step in the reasoning induces a context shift that causes the apparently contradictory claims to occur at different contexts. Attempts at identifying the most promising contextualist account often rely on *timing arguments*, which seek to isolate a step at which the context cannot be claimed to have shifted or must have shifted. The literature contains a number of timing arguments that draw incompatible conclusions about the location of the context shift. I argue that no existing timing arguments succeed. An alternative strategy for assessing contextualist accounts evaluates the plausibility of their explanations of why the context shifts. However, even this strategy yields no clear verdict about which contextualist account is the most promising. I conclude that there are some grounds for optimism and for pessimism about the potential to adequately motivate contextualism.

## Introduction

The Liar sentence *L* in (1a) can be used to produce an apparently valid argument proving both (1b) and (1c):

- *L* = ‘*L* is not true’.
- *L* is not true.
- *L* is true.

*The Liar paradox* threatens the consistency of any theory of the meaning of a language that contains a truth predicate. There has been increasing interest in *contextualist* solutions, which take some step in the reasoning to induce a context shift that causes the apparently contradictory claims to occur and hold at different contexts (see Parsons 1974; Barwise and Etchemendy 1987; Simmons 1993, 2015, 2018; Glanzberg 2001, 2004, 2006; Murzi and Rossi 2018; Burge 1982; Koons 1992). Contextualist approaches have a number of appealing features: they uphold classical logic (cf., Kripke 1975; Field 2008; Priest 1979) and the inferential role of the truth predicate (cf., Tarski 1936), they appear to avoid the ‘revenge’ paradoxes that threaten alternative approaches (see Murzi and Rossi 2018, 2020a, b), and they have an intuitive appeal once it is recognised that the truth values of ordinary sentences often depend on their contexts of use.

For instance, it is easy to imagine a pair of contexts $c_1$ and $c_2$ such that (2a) is true as used at $c_1$ and (2b) is true as used at $c_2$:

- I lost everything in a fire.
- I didn’t lose everything in a fire.

The interpretation of ‘I’ might vary: perhaps Xie (who lost all of her possessions in a fire) is the speaker at $c_1$ and Yuri (who did not lose all of his possessions in a fire) is the speaker at $c_2$. Alternatively, the way in which ‘everything’ is understood might vary: maybe the relevant set of things consists of items inside a particular house (all of which were lost in a fire) at $c_1$, while the relevant set of things includes the speaker’s possessions outside the house (some of which were not lost in a fire) at $c_2$. A contradiction is derivable from (2a) and (2b) only if the context shift is not recognised. Analogously, contextualists claim that the impression that the Liar sentence leads to contradiction vanishes if a context shift between (1b) and (1c) is acknowledged.

It is obvious which expressions are context dependent in (2a) and (2b): indexicals like ‘I’ and quantifier expressions like ‘everything’ are consistently sensitive to their contexts of use. It is less obvious what expressions in the Liar reasoning might be context dependent. A popular strategy is to identify a covert component of a Liar sentence—typically, a quantifier expression ranging over propositions—that connects its interpretation with a context-dependent domain of propositions (see Parsons 1974; Barwise and Etchemendy 1987; Glanzberg 2001, 2004, 2006; Murzi and Rossi 2018).^1^ Similarly, it is obvious what sort of factors cause the context to differ between occurrences of (2a) and (2b): either the speaker has changed, or the set of relevant things (perhaps fixed by the speaker’s intentions) has changed. Yet it is far from obvious what factors could cause the context to shift between occurrences of (1b) and (1c). In order to motivate contextualism, an explanation is required of *when* and *why* the context shifts in the course of the Liar reasoning.

The trouble is that existing contextualist accounts give conflicting explanations. It is important to adjudicate between these accounts because contextualism in general can be well-motivated if and only if some specific contextualist account is plausible. To evaluate existing accounts, arguments are often used that seek to establish that the context cannot coherently be claimed to shift at a certain step, or must shift by a certain step. Such arguments—which I will call ‘*timing arguments*’—evaluate potential locations for the context shift on the basis of their compatibility with the soundness of the contextualist’s version of the Liar reasoning, without recourse to any particular explanation of why the context should shift at that point. This paper aims to show that no existing timing arguments succeed. Section 2 describes existing timing arguments, which are then countered in Sect. 3. A remaining option—attempted in Sect. 4—is to evaluate different contextualist accounts on the basis of the plausibility of their explanations of why the context shifts. I conclude with some grounds for optimism and for pessimism with respect to the project of motivating contextualism.

## Timing arguments

Section 2.1 provides an informal version of the Liar reasoning and sets out Glanzberg’s (2004) contextualist account, along with a timing argument he gives claiming that step (iv) must cause the context shift. Section 2.2 describes Gauker’s (2006) arguments against positing a context shift at any point before step (vi). Section 2.3 sets out Murzi and Rossi’s (2018) arguments opposing the view that the context shifts before step (v), in addition to their proposal that step (v) causes a context shift.

### Glanzberg’s position

According to Glanzberg, to say ‘$\phi$ is true’ is really to say ‘There is some true proposition expressed by $\phi$ in context *c*’ (2004), pp. 32–3). Hence a Liar sentence like (1a) has a logical form that states ‘There is no true proposition expressed by *L* in context *c*’. He represents this logical form as ‘$\lnot \exists p(\textsf {Exp}(L, p) \wedge \textsf {Tr}(p))$’, where ‘$\textsf {Exp}$’ is interpreted as a two-place version of the expression relation that omits reference to contexts, and ‘$\textsf {Tr}$’ is a truth predicate of propositions.^2^ Glanzberg holds that the covert quantifier expression in Liar sentences is affected by its context of use, in a similar way to how overt quantifier expressions in sentences like (2a) and (2b) are. Hence *L* is not true relative to the context in which (1b) is used because the domain contains no true proposition for it to express, whereas *L* is true relative to the context in which (1c) is used because the domain has expanded to include a true proposition for it to express.

According to this position, an informal version of the Liar reasoning is most accurately represented by using an overtly propositional variant $L_p$ of the Liar sentence:^3^

(3) i. $L_p$ = ‘$L_p$ does not express a true proposition’. (A plain fact.)
ii. Suppose that $L_p$ expresses a proposition.
iii. The proposition that $L_p$ expresses is true if and only if it is not true. (By reasoning from (i) and (ii).)
iv. $L_p$ does not express a proposition. (From (ii)–(iii).)
v. $L_p$ does not express a true proposition. (From (iv).)
vi. ‘$L_p$ does not express a true proposition’ expresses a proposition. (From (v) by the principle that if a sentence can be proved from true premises, then it must express a proposition.)
vii. $L_p$ expresses a proposition. (From (i) and (vi).)

The contextualist is committed to the view that step (iv) pertains to a context $c_1$ where there is no proposition for $L_p$ to express, and (vii) occurs in a context $c_2$ where there is a true proposition for $L_p$ to express. Hence a well-motivated contextualist account must identify a context shift somewhere before step (vii), in addition to explaining why the context should shift at the relevant point.

Glanzberg (2004), p. 34) briefly states a timing argument for taking the context to shift between steps (iv) and (v): ‘the truth of [(iv)] requires that there be no proposition for [$L_p$] to express, while the truth of [(v)] requires that there be one’. The idea is that the occurrence of the Liar sentence in step (v) can only be true relative to a context in which the Liar sentence expresses a proposition, even though the inference that the Liar sentence expresses a proposition is not explicitly derived until step (vii). This argument would rule out contextualist accounts that posit a context shift after step (v), such as Murzi and Rossi’s (see Sect. 2.3). This is the only timing argument given by Glanzberg, and the remainder of his work develops an explanation of the context shift.

To explain why the context shifts at the point in question, Glanzberg begins by observing that ‘context provides a running record of [...] what is *salient in a discourse* at a particular point’ (2004, p. 37). This ‘*salience structure*’ is fixed for a given context, so changes of salience structure require changes of context. Salience structures can be affected by the linguistic items used at a context. For example, an occurrence of ‘a wine glass’ in an utterance of (4) causes the salience structure to expand to include a new glass:^4^

(4) I broke a wine glass last night. It was expensive.

Salience structures also affect the interpretation of linguistic items. For instance, ‘it’ is understood to refer to a wine glass in an occurrence of (4) only due to the inclusion of an appropriate item in the salience structure.

Analogously, using a linguistic item that includes ‘express’ in its surface or logical form may expand the salience structure to include the expression relation. Glanzberg (2004, p. 39) proposes that step (iv) has this effect, because it is the first point in the Liar reasoning where ‘express’ occurs without any undischarged assumptions. The addition of the expression relation to the salience structure shifts the context to one where speakers are able to assert propositions concerning semantic relations like the expression relation, in a similar way to how the addition of a wine glass to a salience structure allows speakers to assert propositions about that glass.^5^ The domain of propositions that may be expressed by speakers at the later context thus exceeds the domain for the earlier context.

With the exception of Murzi and Rossi, all other contextualists who take a position on which step causes the context shift—namely, Simmons, Burge and Koons—identify (iv) as this step; although their explanations of the cause diverge significantly from Glanzberg’s. Hence timing arguments that target Glanzberg’s account will also target these other accounts.

### Gauker’s position

In his extended criticism of contextualism, Gauker (2006), p. 403) advocates making the relativity to context explicit in formulations of the Liar reasoning. The reasoning given in (3) may thus be reformulated:

(5) i. $L_p$ = ‘$L_p$ does not express a true proposition in $c_1$’. (A plain fact.)
ii. Suppose that $L_p$ expresses a proposition in $c_1$.
iii. The proposition that $L_p$ expresses is true in $c_1$ if and only if it is not true in $c_1$. (By reasoning from (i) and (ii).)
iv. $L_p$ does not express a proposition in $c_1$. (From (ii)–(iii).)
v. $L_p$ does not express a true proposition in $c_1$. (From (iv).)
vi. ‘$L_p$ does not express a true proposition in $c_1$’ expresses a proposition in $c_2$. (From (v) by some new principle.)
vii. $L_p$ expresses a proposition in $c_2$. (From (i) and (vi).)

Once the context-relativity has been made explicit, Gauker takes a number of timing arguments to emerge against the view that the context shifts at any point between steps (iv) and (vi).

First, Gauker (2006), p. 402) suggests that since (v) follows from (iv) simply by logic, it would be preferable to locate the context shift between (v) and (vi). Presumably, the thought is that the inference from $\phi$’s failure to express a proposition to $\phi$’s failure to express a true proposition (or a false proposition, or a proposition with any other property) is justified by a basic logical principle that is applicable at a single context, and there is no obvious principle that would justify such an inference across distinct contexts.

Gauker additionally argues (pp. 403–4) that the inference of (5)(vi) can no longer be justified. The trouble is that (3)(vi) could be inferred from (3)(v) via the intuitively plausible principle that if a sentence can be proved from true premises, then it must express a proposition. Once the expression of propositions has been explicitly relativised to contexts, a new principle would be required. A plausible new principle would state that if a sentence is provable from premises that are true *in a context* $c_i$, then it must express a proposition *in* $c_i$. Yet Gauker argues that this new principle would allow genuinely contradictory versions of the Liar reasoning to be produced. To illustrate, he stipulates a version of (5) where the reasoning for steps (i) to (v) is carried out in $c_1$, and occurrences of ‘in $c_2$’ in steps (vi) and (vii) are replaced with occurrences of ‘in $c_1$’:

(6) vi. ‘$L_p$ does not express a true proposition in $c_1$’ expresses a proposition in $c_1$. (From (v) by the principle that if a sentence is provable from premises that are true in $c_i$, then it must express a proposition in $c_i$.)
vii. $L_p$ expresses a proposition in $c_1$. (From (i) and (vi).)

The new principle would justify the inference of (6)(vi): the sentence in (v) is provable from premises that are true in $c_1$, so it expresses a proposition in $c_1$. The problem is that (6)(vii) would genuinely contradict (iv). Since the ‘plausible’ new principle can yield a contradiction, Gauker concludes that the principle must be rejected. An alternative new principle would state that if a sentence is provable from premises that are true *in a context* $c_i$, then it must express a proposition *in some other context* $c_j$; yet Gauker deems this principle implausible.^6^ He concludes that no justification can be given for inferring step (5)(vi), which undermines the potential to develop any account that posits a context shift before that step.

### Murzi and Rossi’s position

Murzi and Rossi (2018) present two further arguments against Glanzberg’s account of the context shift, again based on the explicitly context-relative formulation of the Liar reasoning in (5). First, they suggest that, in many cases where the utterance of an expression changes the context, the context must have already changed by the time that expression is interpreted. They make this claim on the basis of occurrences of sentences like (2b) (‘I didn’t lose everything in a fire’), where Yuri’s utterance of (2b) changes the context to one where he is the speaker, and his use of ‘I’ is interpreted relative to the new context. Analogously, if (iv) is the first use of ‘express’ without undischarged assumptions, and this changes the salience structure, ‘then *context should already shift there*’ (Murzi and Rossi 2018, p. 6).

This is not in itself a timing argument: it raises doubts about Glanzberg’s explanation of the way in which the salience of the expression relation induces a context shift, but it does not present soundness-based considerations against postulating a new context at step (iv). Yet a timing argument can be derived from Murzi and Rossi’s first argument for two reasons: their explicit timing arguments (see below) purport to rule out step (iv)’s occurring in a new context, and it is independently difficult to see how any principle would allow step (5)(iv) to be inferred in $c_2$ from the derivation of steps (i)–(iii) in $c_1$.

Murzi and Rossi give timing arguments that hold that the contextualist’s version of the Liar reasoning will fail to be valid if either step (iv) or (v) occurs in the new context. They claim that, in the former case, (5)(iv) would state that $L_p$ does not express a proposition in $c_2$; yet this version of (iv) could not be inferred from the contradiction in line (iii), since it is not the negation of the supposition in line (ii). In the latter case, (5)(v) would state that $L_p$ does not express a true proposition in $c_2$; yet this version of (v) would be non-identical to $L_p$ in line (i), hence the derivation of lines (vi) and (vii) would be blocked and the contextualist thesis that $L_p$ expresses a proposition relative to a later context would lack motivation.

Despite their opposition to Glanzberg’s account of the context shift, Murzi and Rossi are themselves contextualists who assign a similar logical form to the Liar sentence. Their timing arguments against a context shift before either step (iv) or (v) lead them to conclude that the context must shift between steps (v) and (vi). Their explanation for this shift first assumes that for each context *c*, there is some context $c'$ that *extends* *c* (every proposition available to be expressed in *c* is also available in $c'$) *strictly* (*c* does not extend $c'$) (p. 10). Then, they define a principle stating that one can only attribute truth to a proposition expressed by a sentence proved in *c* in a context $c'$ that strictly extends *c*. They formalise this principle as follows, where they use ‘$\vdash ^{c}_S$’ to represent an inference that takes place in context *c* (technically, an inference in a context-relative theory $S_{c}$):

(C-NEC) If $\vdash ^{c}_S \phi$, then $\vdash ^{c'}_S \exists _{c'} p (Exp (\ulcorner \phi \urcorner , p, c') \wedge \mathbf{Tr} (p))$

They argue that C-NEC is independently motivated by observations surrounding reflection principles in arithmetic.^7^

To apply C-NEC, they discuss a version of the explicitly context-relative Liar reasoning slightly different to (5), where steps (vi) and (vii) may be informally presented as follows:

(7) v. $L_p$ does not express a true proposition in $c_1$. (From (5)(iv).)
vi. ‘$L_p$ does not express a true proposition in $c_1$’ expresses a *true* proposition in $c_2$. (From (v) by C-NEC.)
vii. $L_p$ expresses a *true* proposition in $c_2$. (From (5)(i) and (vi).)

The potential for contradiction in (7) then emerges between lines (v) and (vii), rather than between lines (iv) and (vii) as in (5). They claim that this version is more in line with standard presentations of the Liar reasoning.

### Summary of Sect. 2

The current section illustrates the extensive disagreement in the literature about the location of the context shift. That is, Glanzberg argues that it must occur between steps (iv) and (v). Gauker thinks that Glanzberg should locate it between steps (v) and (vi), but he then raises an argument against the potential for a context shift at any point between lines (iv) and (vi). Murzi and Rossi raise further arguments against a context shift before lines (iv) or (v), but propose an account where the context shifts between lines (v) and (vi). It is important to establish whether any of these timing arguments help to identify the location of the context shift.

## Against timing arguments

Sects. 3.1–3.3 challenge all of these timing arguments. I claim that the soundness of the contextualist’s version of the Liar reasoning—including the explicitly context-relative version (5)—is compatible with a context shift between steps (iv) and (v) (as proposed by Glanzberg, Simmons, Burge and Koons) or between steps (v) and (vi) (as proposed by Murzi and Rossi).

### Against Glanzberg’s argument

While Glanzberg’s salience-based explanation of why the context shifts entails that it shifts between steps (iv) and (v), there is an available response to his independent timing argument for such a location of the context shift.

Glanzberg’s argument was that the truth of the sentence in (3)(iv) depends on the absence of a proposition for the Liar sentence to express, whereas the truth of (v) depends on the presence of such a proposition. This is based on the intuitively compelling principle that if a sentence is provably true at a context *c*—such as $L_p$ in the context of step (v)—then it expresses a true proposition at *c*. Yet Murzi and Rossi motivated a principle C-NEC that opposes exactly this view: for a sentence proved at a context *c*, it is only provable at a context $c'$ strictly extending *c* that that sentence expresses a true proposition. Hence the provability of $L_p$ in the context of step (v) does not entail that there is a true proposition for $L_p$ to express at that context.

Murzi and Rossi’s principle might seem counterintuitive, an issue that will be discussed further in Sect. 4.2. Yet the availability of a prima facie viable response to Glanzberg’s timing argument shows that contextualists are not forced to invoke a context shift between steps (iv) and (v).

### Against Gauker’s arguments

Gauker argued that identifying a context shift at any point between steps (5)(iv) and (vi) threatens the validity of the contextualist’s version of the Liar reasoning. A reasonable response claims that two principles would allow the inference of steps (v) and (vi) after a context shift, without entailing the derivability of genuinely contradictory versions of the Liar reasoning.

Gauker’s first argument was that it would be more plausible to identify a context shift between steps (v) and (vi) than between steps (iv) and (v), since (v) appears to be justified by logical principles that are applicable only for a fixed context. However, there are good grounds for thinking that the inference of (5)(v) may follow by standard logic even if the context shifts between (iv) and (v). This can be shown by explicitly including an additional step between (iv) and (v), where all contextualists are likely to accept the provability of this step.

Murzi and Rossi (2018, p. 4) argue that each contextualist theory $S_c$ satisfies the following requirement, which states that for any context *c*, and any context $c'$ where all of the propositions available to be expressed at *c* are available, any sentence provable in *c* will be provable in $c'$:

(EXT) If $\vdash ^{c}_S \phi$, then $\vdash ^{c'}_S \phi$, provided $c'$ extends *c*.

All existing contextualist accounts agree that the sentence in (iv) has been proved relative to $c_1$, and that $c_2$ extends $c_1$; hence EXT entails that the sentence in (iv) is provable at $c_2$. If the occurrence of step (iv) causes the context to shift to $c_2$, then the following step (iv$*$) may be inserted between (5)(iv) and (v):

iv.: $L_p$ does not express a proposition in $c_1$. (From (ii)–(iii).)
iv$$*$$.
$L_p$ does not express a proposition in $c_1$. (from (iv), by EXT.)
v.: $L_p$ does not express a true proposition in $c_1$. (From (iv$*$).)

Crucially, while the context in which (iv) is inferred is $c_1$, the context in which (iv$*$) is inferred is $c_2$.^8^ The basic logical principle envisaged by Gauker may then apply at a single context, in order to justify the inference at $c_2$ of (v) from (iv$*$). Hence the view that step (v) is justified by principles that apply at a fixed context is perfectly compatible with the view that step (iv) causes a context shift.

Gauker’s second argument was that the only plausible new principle that would support the inference of (5)(vi)—that if a sentence is provable from premises that are true in $c_i$, then it must express a proposition in $c_i$—allows the derivation of genuinely contradictory versions of the Liar reasoning. Of course, one response would be to motivate the new principle that Gauker deems implausible—that if a sentence is provable from premises that are true in $c_i$, then it must express a proposition in some other $c_j$—since this principle would not allow the derivation of genuinely contradictory versions. Murzi and Rossi pursue this strategy when they motivate C-NEC (see Sect. 2.3). However, the remainder of the current subsection will focus on a strategy that grants Gauker’s judgement about the implausibility of the latter new principle.

Once we distinguish between the context in which premises are taken to be true and the context in which a conclusion is inferred—which cross-contextual principles like EXT allow to diverge—we can formulate Gauker’s plausible new principle as NP, and compare it with an alternative, NP$*$:

(NP): If a sentence $\phi$ is provable in $c_i$ from premises that are true in $c_i$, then $\phi$ must express a proposition in $c_i$.
(NP$$*$$)
If $\phi$ is provable in $c_i$ from premises that are true in $c_k$ (where either $c_k = c_i$ or cross-contextual principles justify the inference), then $\phi$ must express a proposition in $c_i$.

While Gauker does not comment on the plausibility of a principle like NP$*$, it is a natural generalisation of NP for those who grant the potential for cross-contextual inferences in the course of a single argument: for like NP, the provability of a sentence at a context entails that it expresses a proposition at that same context. Moreover, if line (v) is inferred in $c_2$, then NP$*$ justifies the step (5)(vi) inference that the sentence in line (v) expresses a proposition as used in $c_2$.

It remains to be shown that NP$*$ need not allow the derivation of the genuinely contradictory variant (6) stipulated by Gauker. It is plausible to think that, if a given step in the Liar reasoning causes a context shift, then this step will continue to cause a context shift no matter what stipulations are made about the context of that step or of subsequent steps. This reflects the behaviour of certain natural language expressions. For instance, an occurrence of ‘I broke a wine glass last night’ that shifts the context to one with an expanded salience structure will continue to do so even if the speaker tries to ensure that her subsequent utterances are interpreted relative to the earlier context and salience structure.^9^ Analogously, if step (iv) shifts the context to $c_2$ by expanding the salience structure in the manner that Glanzberg claims (see Sect. 2.1), we would expect this context shift to occur even when attempts are made at stipulating that step (v) occurs in $c_1$ or when subsequent steps mention $c_1$.^10^ If (v) is always inferred at a context $c_2$ distinct from $c_1$, then NP$*$ could never be used to derive the variant of (vi) envisaged by Gauker, where the sentence in (v) is said to express a proposition in $c_1$. NP$*$ could only allow the inference of a version of (vi) stating that the sentence in (v) expresses a proposition in $c_2$.

In sum, a contextualist can coherently uphold the view that (5)(iv)—a sentence denying that $L_p$ expresses a proposition in context $c_1$—is provably true at $c_1$, and that the occurrence of step (iv) causes an obligatory shift of context. A principle like Murzi and Rossi’s EXT then justifies the inference of a previously omitted step (iv$*$), which states the same sentence as (iv) but is inferred at context $c_2$. Simple logic justifies the inference from (iv$*$) of (v), relative to a fixed context. A generalisation of the plausible new principle mentioned by Gauker then allows the derivation of (vi), without allowing a genuinely contradictory version of the Liar reasoning. Hence Gauker’s timing arguments, which target the view that the context shift is induced by any step before (vi) and by step (iv) in particular, can be resisted.

### Against Murzi and Rossi’s arguments

Murzi and Rossi firstly opposed Glanzberg’s explanation of how step (iv) causes a context shift, and secondly argued that the context cannot shift before step (v) is inferred. Their first argument is addressed by considering the different ways in which expressions can affect the context. A response to their second argument then distinguishes the contexts in which the steps of an argument are inferred from the contexts referred to in those steps.

Murzi and Rossi’s first argument was that Glanzberg’s explanation of the reason (iv) induces a context shift would entail that the context had already shifted by step (iv). If correct, their argument requires an advocate of Glanzberg’s explanation to provide and motivate a new principle that would justify the inference of (iv) in $c_2$ from preceding steps inferred in $c_1$. However, the linguistics literature on context change and salience (e.g., Lewis 1979; Heim 1983; Stalnaker 1998, 2002; von Fintel 2008) indicates that expressions need not affect contexts in the manner proposed by Murzi and Rossi’s argument.

As Murzi and Rossi correctly note, an utterance of ‘I’ (or any other expression) yields a context where the utterer is the speaker, and ‘I’ is interpreted as referring to the speaker of the new context. It might therefore be tempting to infer that whenever an utterance changes the context, that utterance is interpreted relative to the new context. Yet a distinction must be drawn between cases where the *act* of making an utterance changes the context and cases where something to do with the *content* of an utterance—whether that is the content itself or associated pragmatic information—changes it. When something to do with the content of an utterance changes the context, the utterance must be interpreted *before* the change of context occurs; although the utterance may well be interpreted relative to a context that has been updated to reflect the fact that the utterance has been made (see Stalnaker 1998, p. 8).^11^ For example, it is something about the *content* of the first clause of (4) (‘I broke a wine glass last night’) that shifts the context to one where a wine glass is salient, not the mere fact that an utterance has been made. Hence the first clause of (4) must occur in, and be interpreted relative to, a prior context that lacks a salient wine glass but has been updated to reflect the current speaker. Similarly, it is something about the content of the sentence in step (iv)—specifically, the effect of interpreting ‘express’—that Glanzberg takes to induce a context shift. Hence if step (iv) causes the context to shift from $c_1$ to $c_2$ by virtue of expanding the salience structure, then (iv) occurs in $c_1$.

A response to Murzi and Rossi’s second argument begins with the observation that their formal theory explicitly distinguishes the context in which a step of an argument is inferred from the contexts of use referred to in steps of an argument. For instance, they represent an inference that takes place *in context* $c'$ stating that *L* expresses a true proposition *in context* *c* as: $\vdash ^{c'}_S \exists _c p (Exp (\ulcorner L \urcorner , p, c) \wedge \mathbf{Tr} (p))$ (2018, p.11). Yet their second argument against Glanzberg’s account overlooks this distinction. Their argument was that, if the context has shifted by step (iv) or (v), then (5)(iv) would state that $L_p$ does not express a proposition in $c_2$ and (5)(v) would state that $L_p$ does not express a true proposition in $c_2$, where this would render the contextualist’s version of the Liar reasoning invalid. Yet it does not follow from the supposition that the context has shifted to $c_2$ by step (iv) or (v) that the contexts mentioned in those steps will become $c_2$. In other words, suitable principles will allow the inference in $c_2$ of sentences stating that $L_p$ does not express a (true) proposition as used in $c_1$. If steps (iv) and (v) will remain as formulated in (5) even if the context has shifted by these steps, then (iv) and (v) would continue to respectively be the negation of the assumption in line (ii) and identical to the formulation of $L_p$ in line (i), preserving the validity of (5).

In sum, the potential for cases where the content of natural language expressions changes the context allows Glanzberg’s proposal to predict that step (iv) will be inferred in $c_1$ while causing step (v) to be inferred in $c_2$. Moreover, if the inference context has shifted to $c_2$ by step (iv) or (v), it does not follow that the contexts mentioned in those steps will become $c_2$. Hence contextualists are free to claim that a step prior to (v) triggers the context shift.

### Summary of Sect. 3

The current section has responded to all timing arguments that have been developed in the literature.^12^ That is, Glanzberg’s claim that step (iv) causes a context shift may be defended from the criticisms of Gauker and Murzi and Rossi; and Murzi and Rossi’s claim that step (v) causes a context shift can be defended from the criticisms of Gauker and Glanzberg. Hence a context shift at any point between steps (iv) and (vi) remains prima facie viable. In other words, we cannot adjudicate between existing contextualist accounts via arguments targeting their views of *when* the context changes. For there just do not seem to be any general, soundness-based considerations that provide good arguments for a particular location of the context shift. An alternative form of adjudication to explore centres on explanations of *why* the context shifts.

## Explanatory arguments

I will call arguments that evaluate a particular explanation of why the context shifts ‘*explanatory arguments*’.^13^ The current section sketches a number of explanatory arguments targeting the accounts given by Glanzberg (in Sect. 4.1) and by Murzi and Rossi (in Sect. 4.2), along with potential responses. I aim to show that there is more scope for the development of promising explanatory arguments than of timing arguments. However, even explanatory arguments provide no straightforward verdicts about which type of account would serve as the most promising contextualist solution to the Liar paradox.

### Targeting Glanzberg’s account

Glanzberg emphasises the independent motivation for, and plausibility of, his explanation of the context shift. He claims (2004, p. 39) that the linguistic motivation for salience structures as a feature of contexts ‘is entirely independent of any considerations of the Liar paradox’, which ‘will help to counter the objection that the contextual approach is *ad hoc* or unmotivated’. Nevertheless, a number of explanatory arguments may be used to target his account.

First, it might be argued that evidence related to the interpretation of pronouns opposes Glanzberg’s claim that step (iv) renders the expression relation salient. Adding an item to the salience structure normally makes it an available referent for subsequent occurrences of pronouns; for example, the use of ‘a wine glass’ in (4) renders a wine glass salient and allows the subsequent occurrence of ‘it’ to be understood to refer to the salient wine glass. Yet occurrences of ‘it’ that follow the sentence from step (iv) of the Liar reasoning often cannot plausibly be understood as referring to the expression relation:

(8) $L_p$ does not express a proposition. ? It is a relation.

However, no account of pronouns takes rendering an item salient to be sufficient to cause any given subsequent pronoun to refer to that item (e.g., see Ariel 2001; Rose 2006; von Heusinger 2006). To resist the current argument, an advocate of Glanzberg’s account need only provide an explanation of why ‘it’ cannot be understood to refer to the expression relation in sentences like (8), and find some continuations where ‘it’ can be understood so.^14^

A second explanatory argument would claim that the widely recognised potential to use indefinite noun phrases to expand the salience structure (e.g., ‘a wine glass’ in (4)) is insufficient to motivate the idea that the use of predicates like ‘express’ expands the salience structure in step (iv) of the Liar reasoning. Occurrences of an expression might affect a salience structure because such an effect is encoded as part of the expression’s meaning, or only due to the interaction of contextual features with particular occurrences.^15^ It would be implausible to hold that occurrences of ‘express’ belong in the first of these categories: for indefinite noun phrases are widely considered unusual in their capacity to systematically introduce new items to salience structures (see Heim 1983; Kamp 1981; Christophersen 1939; Abbott 2008). Yet a theorist who takes ‘express’ to affect salience structures only when it occurs in supportive contexts would need to explain why it always has such an effect in step (iv) of the Liar reasoning; moreover, observations related to indefinite noun phrases and their systematic effects on salience structures could not provide this explanation.

Glanzberg has made some comments relevant to this issue. He claims that one common way to expand salience structures without using an indefinite is ‘to introduce a new term into a discourse, in such a way as to mark its interpretation as salient for the discourse’ (Glanzberg 2004, p. 39). Presumably, he takes ‘express’ to be introduced in this way when it occurs in the first step of the Liar reasoning without undischarged assumptions. Nevertheless, one might wonder whether ‘express’ could ever be introduced in this way if it were to occur in a step with undischarged assumptions.^16^ One might also wonder whether the interpretation of any expression used in a step of a proof without undischarged assumptions is added to the salience structure, or whether terms like ‘express’ have a unique capacity in this respect.^17^ A full explanation of how an occurrence of ‘express’ in the Liar reasoning induces a context shift should address these open issues.

A third explanatory argument observes that the way in which salience structures behave in ordinary discourse seems importantly different from the way that they are supposed to behave in the Liar reasoning. Salience structures can ordinarily only be expanded to include items that are already present in the *background domain* (the items that speakers are able to quantify over at a context). For instance, an occurrence of ‘a wine glass’ causes an item that was already present in the background domain to be added to the salience structure: for speakers at contexts in which (4) is used are presumably able to talk about and quantify over wine glasses that existed on the previous day, even if no glasses are salient at the context. Yet in the Liar reasoning, adding to the salience structure a new relation that was previously *absent from the background domain* is supposed to *add to the background domain* that expression relation and certain new propositions. The view that salience structures can interact with background domains in this way requires motivation, hence observations related to salience structures in ordinary contexts do not suffice to explain the Liar reasoning.

One line of defence would argue that the salience of the expression relation is intended to explain what induces a context shift in the course of the Liar reasoning, as opposed to constituting a full explanation of how new items are added to background domains. Indeed, every contextualist account faces the problem of explaining how a proposition previously absent from the background domain is added to the domain. Arguably, only a contextualist who has provided this full explanation is in a position to wield the third explanatory argument against Glanzberg’s account.

### Targeting Murzi and Rossi’s account

Murzi and Rossi similarly emphasise the independent appeal of their explanation of the context shift. They point out that the context-shifting properties of C-NEC are ‘motivated by well-known facts about reflection principles for arithmetic, and properties of contexts’ (2018, p. 16). Still, several explanatory arguments target their account.

First, their explanation of the context shift commits them to the view that the Liar sentence $L_p$ is provable at $c_1$, but it is only provable that $L_p$ expresses a true proposition at a context like $c_2$ that strictly extends $c_1$. As they acknowledge, this is ‘counterintuitive’ (2018, p. 13). Indeed, when Gauker provides timing arguments against identifying a context shift before step (5)(vi) (see Sect. 2.2), he takes it to be obvious that ‘[w]e cannot maintain that if a sentence $\phi$ is provable from sentences that are true in some context, then $\phi$ must express a proposition in some other context’ (2006, p. 403). An explanation of the context shift that better accords with intuitions would therefore appear preferable to Murzi and Rossi’s, all else being equal.

As a response to this kind of objection, Murzi and Rossi (2018, p. 13) claim that the counterintuitiveness of sentences that fail to express propositions at the context *c* in which they are proved ‘is made up for at the ‘next level’, namely in a context that strictly extends [*c*]’. Moreover, if reflection principles provide sufficiently compelling independent motivation for their view, then we might well be persuaded that our naive intuitions are mistaken.

A second explanatory argument proceeds as follows. Murzi and Rossi claim that, for *any* sentence $\phi$ proved at a context *c*—including ‘unproblematic’ ones like ‘0 = 0’—it can only be proved at a different context $c'$ that $\phi$ expresses a true proposition, where $c'$ strictly extends *c*. In other words, a context shift is required in order to state that any inferred sentence expresses a true proposition. One might think that the counterintuitive view is palatable for unusual sentences like those that produce paradoxes, but cannot plausibly be extended to all sentences.

To defend this aspect of their view, Murzi (2018, fn. 18) claim that this uniform treatment of the Liar sentence and all other theorems is in line with the standard contextualist view, which ‘does not postulate any distinction between ‘problematic’ and ‘unproblematic’ sentences’. Moreover, they are open to the possibility of allowing ‘unproblematic’ sentences to express true propositions at the contexts at which they are proved in future work (Ibid.).

A third explanatory argument begins by noting that contextualists think that the context-shifting step occurs alongside some form of meta-theoretical reflection. Murzi and Rossi take this meta-theoretical reflection to consist of expressing acceptance of the context-relative theory for the initial context *c*, in the sense of adding a reflection principle to this theory (see fn. 7 above). Yet Glanzberg (2015, pp. 238-9) argues that this meta-theoretical reflection is ‘more complicated than merely accepting the correctness of the theory we had’, and is ‘rather noting inadequacies of it, and modifying it’. According to Glanzberg, the Liar reasoning leads us to recognise the inadequacy of the semantics for the context-relative theory for *c*—as opposed to the adequacy of that theory—because the semantics seem to classify the Liar sentence as not true (it is not assigned the value true or false at *c*) and also as true (it correctly describes itself as not true).

A response points out that the type of meta-theoretical reflection associated with the context-shifting step need not be overtly realised as a step in the Liar reasoning. The meta-theoretical reflection might instead be a type of psychological event that accompanies the context-shifting step, and theorists might be ill-equipped to accurately report the nature of this reflection. Hence it might be difficult to establish whether it consists of the acknowledgement of the soundness of the theory associated with the original context or the recognition of the inadequacy of the semantics for that theory. Insofar as Murzi and Rossi are able to motivate the former view, or at least to argue that there is insufficient motivation for the latter view, their theory may withstand this third explanatory argument.

## Conclusion

Adjudicating between different contextualist accounts remains a pressing task in the development of a contextualist response to the Liar paradox. My aim has been to establish that the timing arguments prevalent in the existing literature cannot be used for such a task. A remaining option is to use explanatory arguments instead. I developed a number of explanatory arguments that target two contextualist accounts. Yet the availability of prima facie viable responses to all of these arguments shows that even explanatory arguments do not provide a straightforward means of adjudication.

On one hand, the current paper provides contextualists with grounds for optimism: all existing theories can withstand the timing arguments that target them, in addition to several explanatory arguments. Moreover, the development of explanatory arguments that can adjudicate between accounts remains an open possibility. On the other hand, there are some grounds for pessimism: if no timing arguments succeed, and explanatory arguments require further development, then current theorists are unable to motivate any particular account of when and why the context shifts. Yet an explanation of when and why the context shifts is crucial in order to motivate contextualism as a response to the Liar paradox.

## Footnotes

1 A second strategy is to treat the truth predicate as an overt context-dependent expression (see Burge 1982; Koons 1992; Simmons 1993, 2018). Recent research has centred on the first approach, due to arguments that it produces a more plausible version of contextualism (see Glanzberg 2004, pp. 30-1; Murzi and Rossi 2018, fn. 9).

2 Glanzberg thinks that the reference to a context may be ignored because the only potential source of context dependence is the quantifier expression, which is already explicitly represented. Though Murzi and Rossi use a version of ‘Exp’ that applies to a context argument (see Sect. 2.3).

3 This version is from Gauker 2006, p. 402. Steps (i)–(iv) are often considered to be the basic Liar reasoning, with either steps (v)–(vii) or steps (i)–(vii) described as the ‘*strengthened* Liar reasoning’ (see Parsons 1983, p. 253). For current purposes, I just use the phrase ‘the Liar reasoning’ to refer to steps (i)–(vii) (and their analogues in variants of the reasoning that are given later).

4 The new item added to a salience structure is typically taken to be a *discourse referent* representing a glass, rather than an actual glass (see Karttunen 1969; Heim 1983; Kamp 1981). Following Glanzberg, I will often write as if individuals and relations themselves are added to salience structures.

5 Glanzberg (2006) gives a slightly different explanation of why step (iv) causes the context to shift. He attributes it to the *topicality* of the expression relation, where a topic is ‘what is under discussion at a given point in the discourse’ (p. 56). There is no tension between the two explanations, since salience and topicality are closely related, and the expression relation is plausibly rendered both salient and topical in the course of the Liar reasoning. I focus on Glanzberg’s salience-based explanation in the following.

6 Though Murzi and Rossi (2018) provide a version of this new principle and defend its plausibility; see Sect. 2.3.

7 *Reflection principles* state that the provability of $\phi$ within a theory *S* entails the truth of $\phi$. Such principles are typically interpreted as codifying the soundness of *S*, and are unprovable in minimally strong theories (as a consequence of Löb’s Theorem). One can capture the implicit commitment to reflection principles for *S* by adding them to *S*, but this yields a strictly stronger theory $S'$ that expresses the soundness of *S*. The same reasoning extends to a contextualist theory $S_c$: it cannot be proved in $S_c$ that the provability of $\phi$ at *c* (i.e., within $S_c$) entails that $\phi$ expresses a true proposition in *c*, but it is provable in $S_{c'}$ (where $c'$ strictly extends *c*) that the provability of $\phi$ at *c* entails that $\phi$ expresses a true proposition in $c'$. C-NEC then follows as a consequence.

8 As an anonymous reviewer points out, the fact that the exact same sentence is inferred in distinct steps emphasises how contextualists see the Liar reasoning as something more than a sequence of sentences. Contextualists tend to defend the relativisation of arguments’ steps to contexts either by observing that reflection ‘takes place in real time as we work with and reason about our concepts, and so takes place within contexts’ (Glanzberg 2015, fn.27), or by treating arguments as sequences of sentences within distinct context-relative theories (see Sect. 2.3).

9 A possible exception would be if the speaker immediately utters a continuation like: ‘Actually, there was no wine glass’. Then, although her first utterance would expand the salience structure, her continuation might cause the salience structure to be updated to one identical to that of the initial context. Yet in this (highly marked) scenario, the context still would not revert to the initial one, even if the salience structures ended up identical: for the updated context would include the information that the speaker had issued a pair of bizarre utterances.

10 As discussed in Sect. 4.1, observations related to indefinite noun phrases like ‘a wine glass’ are unlikely to fully predict the effects of expressions in the Liar reasoning on salience structures. Still, a contextualist will presumably think that an expression in the context-shifting step combines with some features of the initial context in order to invariably induce a context shift, rather than that a context shift in the Liar reasoning is optional. Otherwise, contextualism would not be equipped to account for all iterations of the Liar reasoning. While contextualists should explain why the context-shifting step invariably shifts the context, the point relevant to current purposes is that contextualists deny that the context shift can be prevented via stipulation.

11 This picture is complicated by *accommodation*, where information that is required for the felicity of an utterance can be added to the context before the interpretation process is complete, causing the utterance to be assigned content relative to the new context (see Stalnaker 1998, pp. 9–10). For expressions that introduce new items to salience structures, accommodation of a salience structure with the new item would be unlikely because it would normally produce *infelicity*. As illustrated by the second clause of (i), it is generally infelicitous to use an indefinite phrase (rather than an expression like ‘it’ or ‘the wine glass’) in an attempt to talk about an item already in the salience structure:

(i) I broke a wine glass last night. ? A wine glass was expensive.

If the first clause of occurrences of (4) and of (i) were to be interpreted relative to a salience structure that already contained the wine glass broken by the speaker last night, then the first clause should be similarly infelicitous.

12 There is one timing argument that I have refrained from challenging (and, indeed, might be inclined to grant): doubts about the potential to provide a new principle that would justify the inference of (iv) in $c_2$ from preceding steps inferred in $c_1$ would constitute a timing argument against a context shift before step (iv) (see the discussion of Murzi and Rossi’s first argument in Sect. 2.3). Still, I opposed Murzi and Rossi’s argument that Glanzberg is committed to the view that (iv) occurs at a new context (see Sect. 3.3), and I do not know of anyone in the literature who accepts such a view. Hence this timing argument would fail to rule out any existing contextualist proposals.

13 Of course, effective explanatory arguments will often entail a particular location for the context shift. For example, a convincing argument in favour of Murzi and Rossi’s explanation of why the context shifts would entail that the context must shift between steps (v) and (vi), since this is a consequence of their explanation and thus a requirement for the soundness of their version of the reasoning (I thank Julien Murzi for emphasising this point). Such an argument would still not be classified as a ‘timing argument’: timing arguments evaluate locations for the context shift purely on the basis of compatibility with the soundness of the contextualist’s Liar reasoning, without recourse to any particular explanation of why the context shifts. By claiming that timing arguments do not adjudicate between existing contextualist accounts, I am claiming that adjudication requires recourse to explanations of why the context shifts.

14 Such an explanation might draw on the well-known preference to understand a pronoun to refer to an item determined by a prior expression in a syntactically parallel position (e.g., see Chambers and Smyth 1998). Since ‘it’ is in subject position in (8), there is a preference to treat the subject position ‘$L_p$’ as its antecedent. Indeed, (i) gives a continuation where it seems natural to interpret ‘it’ as referring to expressing a proposition or standing in the expression relation:

(i) $L_p$ does not express a proposition. Other sentences do it easily.

15 For instance, an occurrence of ‘All of this talk of wine has made me think about *cheese*’ might render cheese salient. Yet it is presumably not encoded in the meaning of ‘cheese’ that every utterance of the expression will add new items to the salience structure (cf. the indefinite determiner ‘a(n)’).

16 Here is one argument that might be developed in order to defend a negative answer. If we embed an expression that systematically adds items to salience structures under a supposition or other modal, then it is seemingly prevented from adding items to ‘global’ salience structures (i.e., for the entire context, rather than the ‘local’ context introduced by the supposition). For instance, the occurrence of ‘it’ in the second clause of (i) cannot naturally be understood to refer to a newly salient glass:

(i) (Suppose / Perhaps / Possibly) I broke a wine glass last night. ? It was expensive.

If ‘express’ is similar to indefinite noun phrases in this respect, and if undischarged assumptions are similar to suppositions, then one might infer that ‘express’ does not add the expression relation to the salience structure for the ‘global’ context when it occurs in a step with undischarged assumptions.

17 Perhaps the idea that terms like ‘express’ are more likely to affect salience structures than other expressions used in steps without undischarged assumptions could be defended by claiming that ‘reflection on the semantics of a language [...] can overtly change the topic’ (Glanzberg 2015, p. 220).
